# Association of marital status with cognitive function in Chinese hypertensive patients: a cross-sectional study

**DOI:** 10.1186/s12888-022-04159-9

**Published:** 2022-07-27

**Authors:** Si Shen, Jianduan Cheng, Junpei Li, Yanyou Xie, Li Wang, Xinlei Zhou, Wei Zhou, Lingjuan Zhu, Tao Wang, Jianglong Tu, Huihui Bao, Xiaoshu Cheng

**Affiliations:** 1 Department of Cardiovascular, the Second Affiliated Hospital of Nanchang University, No. 1 Minde Road, Nanchang, 330006 Jiangxi China; 2 Wuyuan Hospital of Traditional Chinese Medicine, Wuyuan, China; 3 Center for Prevention and Treatment of Cardiovascular Diseases, the Second Affiliated Hospital of Nanchang University, Nanchang, China; 4 Department of Neurology, the Second Affiliated Hospital of Nanchang University, No. 1 Minde Road, Nanchang, 330006 Jiangxi China

**Keywords:** Cognitive function, Marital status, Unmarried, Chinese adapted MMSE (CAMSE), Hypertensive patients

## Abstract

**Purpose:**

The aim of this study was to evaluate the association of marital status with cognitive function and to examine the potential effect modifiers in Chinese hypertensive populations.

**Methods:**

A total of 9,525 adult Chinese hypertensive patients were enrolled in this cross-sectional study. Cognitive function, as the dependent variable in our study, was assessed by the Chinese adapted Mini-Mental State Examination (CAMSE). We adjusted for potential confounding factors in multiple linear regression models to examine the relationship of marital status with cognitive function. In addition, we divided the population according to sex to explore whether there were sex-specific differences.

**Results:**

Among the 9,525 study participants, the mean (SD) age for men was 63.5 (10.3) years, and the mean MMSE score was 24.9 ± 5.0, whereas for women, the mean (SD) age was 63.8 (9.3) years, and the mean MMSE score was 19.4 ± 6.4. Unmarried persons had lower scores on the MMSE and lower subscores in each of the cognitive domains. A stronger correlation between marital status and a lower MMSE score was statistically significant in men (unmarried men: β = -1.55; 95% CI: -1.89, -1.21) but not women (unmarried women: β = -0.22; 95% CI: -0.56, 0.12; p interaction = 0.006). Compared to men who were widowed or divorced, never married men were more likely to have lower MMSE scores (β = -2.30, 95% CI -3.10,—1.50; *p* < 0.001).

**Conclusions:**

Our study demonstrated that being unmarried is an extremely important but neglected social risk factor for cognitive function. Sex was a strong effect modifier: being unmarried was correlated with a higher risk of cognitive decline than being married in Chinese hypertensive men, especially among older men, but this correlation was not observed among women. Moreover, never married men showed poorer cognitive function than those who were divorced or widowed.

**Supplementary Information:**

The online version contains supplementary material available at 10.1186/s12888-022-04159-9.

## Introduction

At present, the proportion of the world’s ageing population is high, and this proportion will continue to increase over time [1]. With the ageing of the population, the prevalence of dementia is increasing year by year, which places a heavy economic burden on patients, their families, and society [2]. In Western countries, Alzheimer's disease is the most frequent form of dementia and the leading cause of disability [3]. The number of Americans suffering from Alzheimer's disease is expected to rise to 13.8 million by mid-century, due in large part to the ageing of the baby boomer generation [2]. China also has a large population of people with cognitive impairment. Preliminary estimates show that 15.07 million people over the age of 60 in China have dementia, and the overall prevalence of dementia is 6.0% [4]. This poses a substantial challenge for policymakers, health care professionals and family members. Therefore, interventions are needed to preserve people’s cognitive function.

Over the past decade, the divorce rate in China has been rising. In 2016, there were 4.16 million divorced couples in China [5]. Moreover, under the influence of traditional attitudes towards a preference for sons, there is a serious imbalance between the number of men and women in China [6]. All these factors have led to a large number of unmarried people of marriageable age in China. Most studies have reported an association between marital status and health, while divorce and widowhood have deleterious effects on health, including self-rated health, cardiovascular health, and the risk of inflammation [7, 8].

Previous articles have stated that divorced and widowed older adults are prone to cognitive dysfunction [9, 10]. However, the number of such studies conducted in the Chinese population is relatively small. Recent studies have suggested that women are more psychologically and physically affected by marital stress than men [11]. Evidence on differences by sex in the relationship between marital status and cognitive function, however, is still inconclusive. A study from the United States found that sex did not change the association between marital status and cognitive impairment [9]. Recently, Xu et al. focused on older Chinese adults and found that, among older Chinese men, single men have worse cognitive function than their married peers, but this was not found among women [12]. Unfortunately, because of the limited number of participating residents, this conclusion must still be experimentally confirmed.

Beyond this, there is evidence suggesting that hypertension has emerged as a leading cause of cognitive impairment [13, 14]. Hypertension is very common in China, and its prevalence is increasing annually [15]. We believe that it is of great social significance to explore the relationship between marital status and cognitive function in high-risk groups with cognitive impairment. To the best of our knowledge, there have been no studies on the relationship between marital status and cognitive function in people with hypertension. In summary, given that there are a large number of people with dementia and unmarried people of marriageable age in China, we believe that conducting this study was necessary. Our study aimed to examine the relationship between marital status and cognitive function in a Chinese hypertensive population and further explore the possible effect modifying factors, which will help to improve the screening of people at high risk of cognitive decline.

## Methods

### Participants

**Fig. 1:**
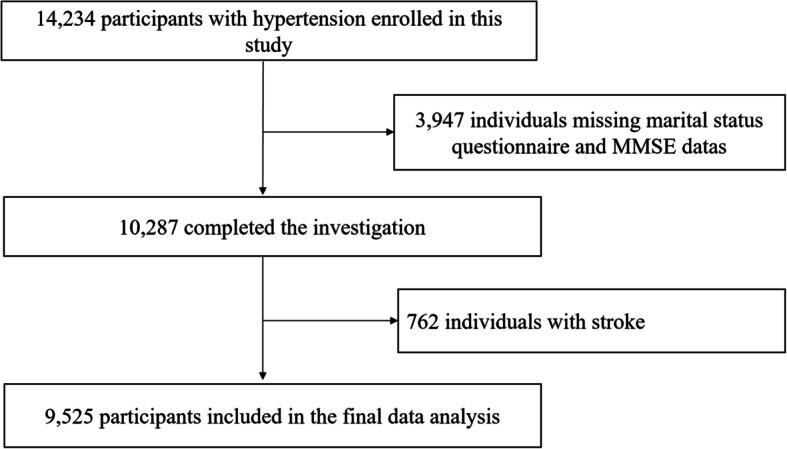
Flow chart of the study participant

The data of this study were obtained from the China H-type Hypertension Registry study (registration number: ChiCTR1800017274). Briefly, this study was a real-world, observational study designed to investigate the prevalence and treatment of hypertension in China and to assess the factors associated with its prognosis. Eligible study participants were Chinese men and women who were aged 18 years or older with hypertension, defined as a measured systolic blood pressure (SBP) ≥ 140 mm Hg and/or a diastolic blood pressure (DBP) ≥ 90 mm Hg (the mean of three measurements, taken after the participants sat quietly for 5 min) and/or a self-reported history of hypertension and/or taking antihypertensive medication at the time of recruitment [15]. The exclusion criteria were as follows: (1) Participants who failed to provide informed consent due to psychological or nervous system impairment; and (2) After evaluation, participants for whom follow-up could not be completed according to the study requirements. Ultimately, 14,234 hypertensive participants from Wuyuan County, Jiangxi Province, China, between March 2018 to August 2018 were recruited for our study. A total of 3947 study participants were excluded due to a lack of marital status and MMSE score data. Given the possible impact of stroke on cognitive function [16–18], we excluded stroke patients (*n* = 762). A total of 9,525 hypertensive participants were included in the final analysis (Fig. 1).

The study was approved by the Ethics Committee of the Biomedical Institute of Anhui Medical University (NO. CH1059) and the Ethics Committee of the Second Affiliated Hospital of Nanchang University (NO. 2018019). All study patients were admitted for enrolment in this study after being informed; informed consent was obtained from the patients themselves or from the legal guardians of illiterate patients.

### Data collection

The health questionnaires were conducted by researchers with professional training. Demographic information collected through questionnaires included sex, age, lifestyle (such as smoking status and alcohol drinking status), medical history (such as coronary heart disease, diabetes, and stroke), and medication (such as hypoglycaemic drugs, lipid-lowering drugs, antihypertensive drugs, and antiplatelet drugs). Our questionnaire also included some questions about other information, which are described as follows: “How do you feel about your standard of living in the local area?” Answers were selected from “1. Better; 2. General; or 3. Poorer.” “How do you feel about your labour intensity in your daily work?” Answers were selected from “1. Light; 2. Medium; or 3. Heavy.” “Does your major occupation and daily life make you feel stressed?” Answers were selected from “1. Rarely; 2. Sometimes; or 3. Always.” Some anthropometric measures (e.g., height, weight, blood pressure) were also collected. Fasting venous blood was collected from all study patients the next day after one night of fasting. The blood samples were immediately frozen and sent to the Biaojia Biotechnology laboratory for analysis in Shenzhen, China. All laboratory measurements met a standardization and certification program. The estimated glomerular filtration rate (eGFR) was formulated using the chronic kidney disease-epidemiology consortium equation [19] (CKD-EPI) rather than the change of food in the renal disease (MDRD) equation. In this study, diabetes was defined as a fasting blood glucose level ≥ 7.0 mmol/L and/or the use of glucose-lowering drugs and/or a self-reported history of diabetes [20].

### Cognitive assessment and marital status

Our study used the Chinese version of the Chinese adapted MMSE (CAMSE) test as a measure of overall cognitive function [21]. The total MMSE score ranges from 0 to 30, and higher values denote better cognitive functioning. The test included five major cognitive domains: orientation, immediate memory, attention and computation, recall, and language. Most of the questions in the MMSE scale could be translated and used directly in this test. The repeated phrase "No ifs, ands or buts" was replaced by “Forty-four stone lions” due to the lack of a suitable Chinese equivalent [22].

Information about the marital status of the study participants was obtained by direct questioning. Four categories are defined in our questionnaire: never married, married, divorced, and widowed. Marital status was assessed by the following question: “What is your current marital status? You can choose to answer married, never married, divorced, or widowed.”

### Covariates

The covariates included in our data analysis were sex (sex was adjusted only in the total sample), age, education level, systolic blood pressure (SBP), diastolic blood pressure (DBP), body mass index (BMI), homocysteine, total cholesterol, triglycerides, high-density lipoprotein cholesterol (HDL-C), low-density lipoprotein cholesterol (LDL-C), eGFR, diabetes, coronary heart disease (CHD), standard of living, labour intensity, stress, sleep duration, use of antihypertensive drugs, smoking status, and alcohol drinking status.

### Statistical analysis

Individuals were stratified into two groups according to whether they were male or female. Continuous variables are shown as the mean ± standard deviation, and categorical variables are presented as frequencies and percentages (%). The population characteristics were described by sex classification and different categories of marital status to explore the distribution of each interval. Marital status had two categories: married or unmarried (never married, divorced, or widowed). With marital status as the independent variable and cognitive function as the dependent variable, multiple linear regression analysis was performed to obtain the regression coefficient (β) and 95% confidence interval (CI) of the association between marital status and cognitive function. The covariates in our study included traditional or suspected risk factors for cognitive function and potential confounding factors that affected estimates that were individually changed by more than 10%. In this study, three models were constructed based on clinical experience and previous relevant literature: Model 1, with no adjustments for any covariates; Model 2, adjusted only for sex (sex was adjusted only in the total sample), age, and education level; and Model 3, adjusted for sex (sex was adjusted only in the total sample), age, education level, SBP, DBP, BMI, homocysteine, total cholesterol, triglycerides, HDL-C, LDL-C, eGFR, diabetes, coronary heart disease, standard of living, labour intensity, stress, sleep duration, use of antihypertensive drugs, smoking status, and alcohol drinking status. Possible modifications of the association between marital status and cognitive function were assessed, including the variables of age, BMI, education level, control of blood pressure, current smoking, current drinking, homocysteine, HDL-C, LDL-C, and eGFR. A two-tailed *p* value less than 0.05 was considered to be statistically significant.

Only a two-sided *p* value < 0.05 was considered statistically significant. Rstudio version 1.3.1093 (https://download1.rstudio.org) and Empower version 3.4.3 (https://www.empowerstats.com; X&Y Solutions, Inc) were used in all data analyses.

## Results

### Baseline characteristics of the study participants

A total of 9,525 hypertensive participants without stroke were included in the final analysis. The distributions of the study participants’ baseline characteristics according to sex and marital status (married and unmarried) are presented in Table 1. In the total sample, a higher percentage of unmarried individuals was women. In addition, people who were unmarried were more likely to have lower MMSE scores. In the men group, unmarried participants had higher values for age, current smoking, and homocysteine, and lower values for BMI, DBP, current drinking, MMSE scores, triglycerides, eGFR, and glucose-lowering drug use. In the women group, unmarried participants were associated with higher values for age, SBP, current smoking, homocysteine, HDL-C, coronary heart disease, and antihypertensive drug use and lower values for BMI, DBP, MMSE scores, triglycerides, eGFR and lipoprotein-lowering drug use. The mean (SD) age for men was 63.5 (10.3) years, and the mean MMSE score was 24.9 ± 5.0, whereas for women, the mean (SD) age was 63.8 (9.3) years, and the mean MMSE score was 19.4 ± 6.4. There was a higher proportion of illiteracy in females than males (58.0% vs. 13.2%). Furthermore, widowed participants had higher values for age and lower MMSE scores (Table S1).

**Table 1:** Baseline characteristics of study participants by sex and marital status

	Marital status (Total)				Marital status (Male)				Marital status (Female)			
Characteristics	Total	married	unmarried	*P*-value	Total	married	unmarried	*P*-value	Total	married	unmarried	*P*-value
Number	9525	7698	1827		4570	3960	610		4955	3738	1217	
Age,years	63.7 ± 9.8	62.1 ± 9.3	70.4 ± 9.0	< 0.001	63.5 ± 10.3	62.7 ± 10.0	69.0 ± 10.4	< 0.001	63.8 ± 9.3	61.4 ± 8.4	71.1 ± 8.2	< 0.001
BMI,kg/m^2^	23.6 ± 3.5	23.8 ± 3.5	22.7 ± 3.5	< 0.001	23.4 ± 3.5	23.6 ± 3.5	22.3 ± 3.3	< 0.001	23.8 ± 3.6	24.1 ± 3.5	22.9 ± 3.5	< 0.001
SBP,mmHg	147.2 ± 17.5	146.8 ± 17.2	149.0 ± 18.4	< 0.001	145.2 ± 17.4	145.2 ± 17.3	145.1 ± 18.1	0.979	149.1 ± 17.4	148.6 ± 17.0	151.0 ± 18.2	< 0.001
DBP,mmHg	89.0 ± 10.8	89.8 ± 10.6	85.5 ± 10.9	< 0.001	90.2 ± 11.1	90.7 ± 10.9	87.0 ± 11.3	< 0.001	87.9 ± 10.4	88.9 ± 10.1	84.8 ± 10.6	< 0.001
Current smoking, N (%)	2498 (26.2)	2054 (26.7)	444 (24.3)	0.038	2208 (48.3)	1884 (47.6)	324 (53.1)	0.011	290 (5.9)	170 (4.5)	120 (9.9)	< 0.001
Current drinking, N (%)	2132 (22.4)	1847 (24.0)	285 (15.6)	< 0.001	1876 (41.1)	1652 (41.7)	224 (36.7)	0.020	256 (5.2)	195 (5.2)	61 (5.0)	0.780
MMSE	22.1 ± 6.4	22.8 ± 6.1	18.7 ± 6.6	< 0.001	24.9 ± 5.0	25.4 ± 4.6	21.7 ± 6.1	< 0.001	19.4 ± 6.4	20.1 ± 6.3	17.2 ± 6.4	< 0.001
Laboratory results												
Homocysteine,umol/L	17.9 ± 11.1	17.6 ± 11.0	18.9 ± 11.3	< 0.001	20.2 ± 13.6	19.9 ± 13.5	22.6 ± 14.3	< 0.001	15.7 ± 7.4	15.2 ± 6.9	17.0 ± 8.8	< 0.001
Total cholesterol,mmol/L	5.1 ± 1.1	5.1 ± 1.1	5.1 ± 1.1	0.078	4.9 ± 1.1	4.9 ± 1.1	4.9 ± 1.0	0.182	5.3 ± 1.1	5.3 ± 1.1	5.3 ± 1.1	0.968
Triglyceride,mmol/L	1.8 ± 1.3	1.9 ± 1.4	1.6 ± 1.0	< 0.001	1.7 ± 1.3	1.7 ± 1.3	1.4 ± 1.0	< 0.001	2.0 ± 1.3	2.1 ± 1.4	1.7 ± 1.0	< 0.001
LDL,mmol/L	2.9 ± 0.8	2.9 ± 0.8	2.9 ± 0.8	0.912	2.8 ± 0.8	2.8 ± 0.8	2.8 ± 0.7	0.070	3.1 ± 0.8	3.1 ± 0.8	3.0 ± 0.8	0.210
HDL,mmol/L	1.5 ± 0.4	1.5 ± 0.4	1.6 ± 0.4	< 0.001	1.5 ± 0.4	1.5 ± 0.4	1.5 ± 0.4	0.086	1.5 ± 0.4	1.5 ± 0.4	1.6 ± 0.4	< 0.001
eGFR,ml/min/1.73m^2^	86.3 ± 19.6	87.9 ± 19.1	79.8 ± 20.2	< 0.001	84.8 ± 20.1	85.6 ± 19.9	79.6 ± 20.3	< 0.001	87.8 ± 18.9	90.3 ± 17.8	79.9 ± 20.1	< 0.001
Education,N(%)				< 0.001				< 0.001				< 0.001
Illiteracy	3477 (36.5)	2466 (32.0)	1011 (55.3)		605 (13.2)	442 (11.2)	163 (26.7)		2872 (58.0)	2024 (54.1)	848 (69.7)	
Primary school	4023 (42.2)	3363 (43.7)	660 (36.1)		2335 (51.1)	1982 (50.1)	353 (57.9)		1688 (34.1)	1381 (36.9)	307 (25.2)	
Middle school and above	2025 (21.3)	1869 (24.3)	156 (8.5)		1630 (35.7)	1536 (38.8)	94 (15.4)		395 (8.0)	333 (8.9)	62 (5.1)	
Standard of living, N (%)				< 0.001				< 0.001				< 0.001
Better	1246 (13.1)	1003 (13.0)	243 (13.3)		667 (14.6)	597 (15.1)	70 (11.5)		579 (11.7)	406 (10.9)	173 (14.2)	
General	6491 (68.1)	5369 (69.7)	1122 (61.4)		3113 (68.1)	2751 (69.5)	362 (59.3)		3378 (68.2)	2618 (70.0)	760 (62.4)	
Poorer	1788 (18.8)	1326 (17.2)	462 (25.3)		790 (17.3)	612 (15.5)	178 (29.2)		998 (20.1)	714 (19.1)	284 (23.3)	
Labour intensity, N (%)				< 0.001				< 0.001				< 0.001
Light	5310 (55.7)	4129 (53.6)	1181 (64.6)		2484 (54.4)	2092 (52.8)	392 (64.3)		2826 (57.0)	2037 (54.5)	789 (64.8)	
Medium	2219 (23.3)	1895 (24.6)	324 (17.7)		1142 (25.0)	1027 (25.9)	115 (18.9)		1077 (21.7)	868 (23.2)	209 (17.2)	
Heavy	1996 (21.0)	1674 (21.7)	322 (17.6)		944 (20.7)	841 (21.2)	103 (16.9)		1052 (21.2)	833 (22.3)	219 (18.0)	
Stress, N(%)				0.957				0.222				0.017
Rarely	6408 (67.3)	5182 (67.3)	1226 (67.1)		3317 (72.6)	2892 (73.0)	425 (69.7)		3091 (62.4)	2290 (61.3)	801 (65.8)	
Sometimes	2294 (24.1)	1854 (24.1)	440 (24.1)		932 (20.4)	795 (20.1)	137 (22.5)		1362 (27.5)	1059 (28.3)	303 (24.9)	
Always	823 (8.6)	662 (8.6)	161 (8.8)		321 (7.0)	273 (6.9)	48 (7.9)		502 (10.1)	389 (10.4)	113 (9.3)	
Sleep duration, h, N (%)				< 0.001				< 0.001				< 0.001
< 5	395 (4.1)	290 (3.8)	105 (5.7)		153 (3.3)	124 (3.1)	29 (4.8)		242 (4.9)	166 (4.4)	76 (6.2)	
≥ 5 to < 8	4958 (52.1)	4096 (53.2)	862 (47.2)		2250 (49.2)	1999 (50.5)	251 (41.1)		2708 (54.7)	2097 (56.1)	611 (50.2)	
≥ 8	4172 (43.8)	3312 (43.0)	860 (47.1)		2167 (47.4)	1837 (46.4)	330 (54.1)		2005 (40.5)	1475 (39.5)	530 (43.5)	
History of disease												
Diabetes, N (%)	1726 (18.1)	1403 (18.2)	323 (17.7)	0.586	721 (15.8)	633 (16.0)	88 (14.4)	0.326	1005 (20.3)	770 (20.6)	235 (19.3)	0.331
CHD, N (%)	533 (5.6)	407 (5.3)	126 (6.9)	0.007	274 (6.0)	232 (5.9)	42 (6.9)	0.320	259 (5.2)	175 (4.7)	84 (6.9)	0.003
Medication use, N (%)												
Antihypertensive drugs	5798 (60.9)	4617 (60.0)	1181 (64.6)	< 0.001	2752 (60.2)	2380 (60.1)	372 (61.0)	0.678	3046 (61.5)	2237 (59.8)	809 (66.5)	< 0.001
Glucose-lowering drugs	447 (4.7)	368 (4.8)	79 (4.3)	0.407	176 (3.9)	163 (4.1)	13 (2.1)	0.018	271 (5.5)	205 (5.5)	66 (5.4)	0.935
Lipoprotein-lowering drugs	258 (2.7)	219 (2.8)	39 (2.1)	0.093	109 (2.4)	96 (2.4)	13 (2.1)	0.659	149 (3.0)	123 (3.3)	26 (2.1)	0.041

Data are expressed as mean ± SD and numbers (percentage) as appropriate

*Abbreviations*: *BMI* Body mass index, *SBP* Systolic blood pressure, *DBP* Diastolic blood pressure, *MMSE* Mini‐Mental State Examination, *LDL-C* Low-density lipoprotein cholesterol, *HDL-C* High-density lipoprotein cholesterol, *eGFR* estimated glomerular filtration rate, *CHD* Coronary heart disease

### Association of marital status with cognitive function in hypertensive patients

Table 2 shows the results of multiple linear regression of the relationship between marital status and cognitive function in patients with hypertension. In adjusted Model 3, participants who were never married (β = -2.18, 95% CI -3.06, -1.30; *P* < 0.001) and widowed (β = -0.81, 95% CI -1.06, -0.56; *P* < 0.001) had lower MMSE scores than those who were married. Because of the small sample size, we combined widowed, divorced and never married participants and collectively referred to these as unmarried participants. We still observed that unmarried people (β = -0.87, 95% CI -1.10, -0.63; *P* < 0.001) had lower MMSE scores than married people. Next, stratified analyses by sex were performed to evaluate sex differences. In contrast, the association between being unmarried and MMSE scores was statistically significant in men (β = -1.55, 95% CI -1.89, -1.21; *P* < 0.001) but not in women (β = -0.22, 95% CI -0.56,0.12; *P* = 0.213). Additionally, we were surprised to find differences in the MMSE scores between different people even if they were all unmarried. Compared to participants who were widowed or divorced, never married men were more likely to have lower MMSE scores (β = -2.30, 95% CI -3.10,—1.50; *p* < 0.001).

**Table 2:** Regression coefficients (95% CIs) of MMSE according to marital status

			Model 1		2Model 2		Model 3	
Marital status	N	Mean + SD	β (95% CI)	*p* value	β (95% CI)	*p* value	β (95% CI)	*p* value
Total								
married	7698	22.8 ± 6.1	Ref		Ref		Ref	
never married	96	21.8 ± 6.3	-1.03 (-2.28, 0.21)	0.104	-2.48 (-3.37, -1.60)	< 0.001	-2.18 (-3.06, -1.30)	< 0.001
divorced	52	24.2 ± 5.1	1.37 (-0.32, 3.05)	0.112	0.07 (-1.12, 1.27)	0.906	0.17 (-1.01, 1.35)	0.778
widowed	1679	18.4 ± 6.6	-4.48 (-4.81, -4.15)	< 0.001	-0.87 (-1.12, -0.62)	< 0.001	-0.81 (-1.06, -0.56)	< 0.001
Marital status								
married	7698	22.8 ± 6.1	Ref		Ref		Ref	
unmarried	1827	18.7 ± 6.6	-4.13 (-4.45, -3.82)	< 0.001	-0.95 (-1.19, -0.70)	< 0.001	-0.87 (-1.10, -0.63)	< 0.001
Male								
married	3960	25.4 ± 4.6	Ref		Ref		Ref	
never married	92	21.9 ± 6.4	-3.56 (-4.56, -2.56)	< 0.001	-2.70 (-3.50, -1.90)	< 0.001	-2.30 (-3.10, -1.50)	< 0.001
divorced	37	24.6 ± 4.5	-0.85 (-2.41, 0.72)	0.288	-0.06 (-1.32, 1.19)	0.920	0.01 (-1.22, 1.25)	0.982
widowed	481	21.5 ± 6.0	-3.98 (-4.44, -3.52)	< 0.001	-1.63 (-2.01, -1.24)	< 0.001	-1.53 (-1.91, -1.15)	< 0.001
Marital status								
married	3960	25.4 ± 4.6	Ref		Ref		Ref	
unmarried	610	21.7 ± 6.1	-3.73 (-4.14, -3.31)	< 0.001	-1.70 (-2.04, -1.36)	< 0.001	-1.55 (-1.89, -1.21)	< 0.001
Female								
married	3738	20.1 ± 6.3	Ref		Ref		Ref	
never married	4	20.2 ± 3.2	0.15 (-6.01, 6.32)	0.961	-0.17 (-4.85, 4.52)	0.945	0.54 (-4.77, 5.84)	0.843
divorced	15	23.3 ± 6.5	3.17 (-0.02, 6.36)	0.051	-0.26 (-2.69, 2.16)	0.833	-0.12 (-2.50, 2.27)	0.924
widowed	1198	17.1 ± 6.4	-2.97 (-3.38, -2.56)	< 0.001	-0.28 (-0.63, 0.07)	0.112	-0.22 (-0.56, 0.12)	0.208
Marital status								
married	3738	20.1 ± 6.3	Ref		Ref		Ref	
unmarried	1217	17.2 ± 6.4	-2.89 (-3.29, -2.48)	< 0.001	-0.28 (-0.63, 0.06)	0.110	-0.22 (-0.56, 0.12)	0.213

Model 1 was adjusted for none. Model 2 was adjusted for sex (sex was adjusted only in the total sample), age, and education. Model 3 was adjusted for sex (sex was adjusted only in the total sample), age, education, SBP, DBP, BMI, homocysteine, total cholesterol, triglyceride, HDL-C, LDL-C, eGFR, diabetes, coronary heart disease, standard of living, labour intensity, stress, sleep duration, antihypertensive drugs, smoking status, alcohol drinking status

We reported the association between marital status and scores on the following MMSE subscores: orientation, immediate memory, attention and computation, recall, and language. Tables S2 to S6 show that, compared with those who were married, those who were not married were more likely to have lower MMSE subscores. Then, we further grouped the population by sex. Compared with married men, unmarried men had lower scores for all MMSE subscores. However, in the women group, unmarried persons seemed to score lower than those who were married only on the immediate memory test.

### Subgroup analysis

**Fig. 2:**
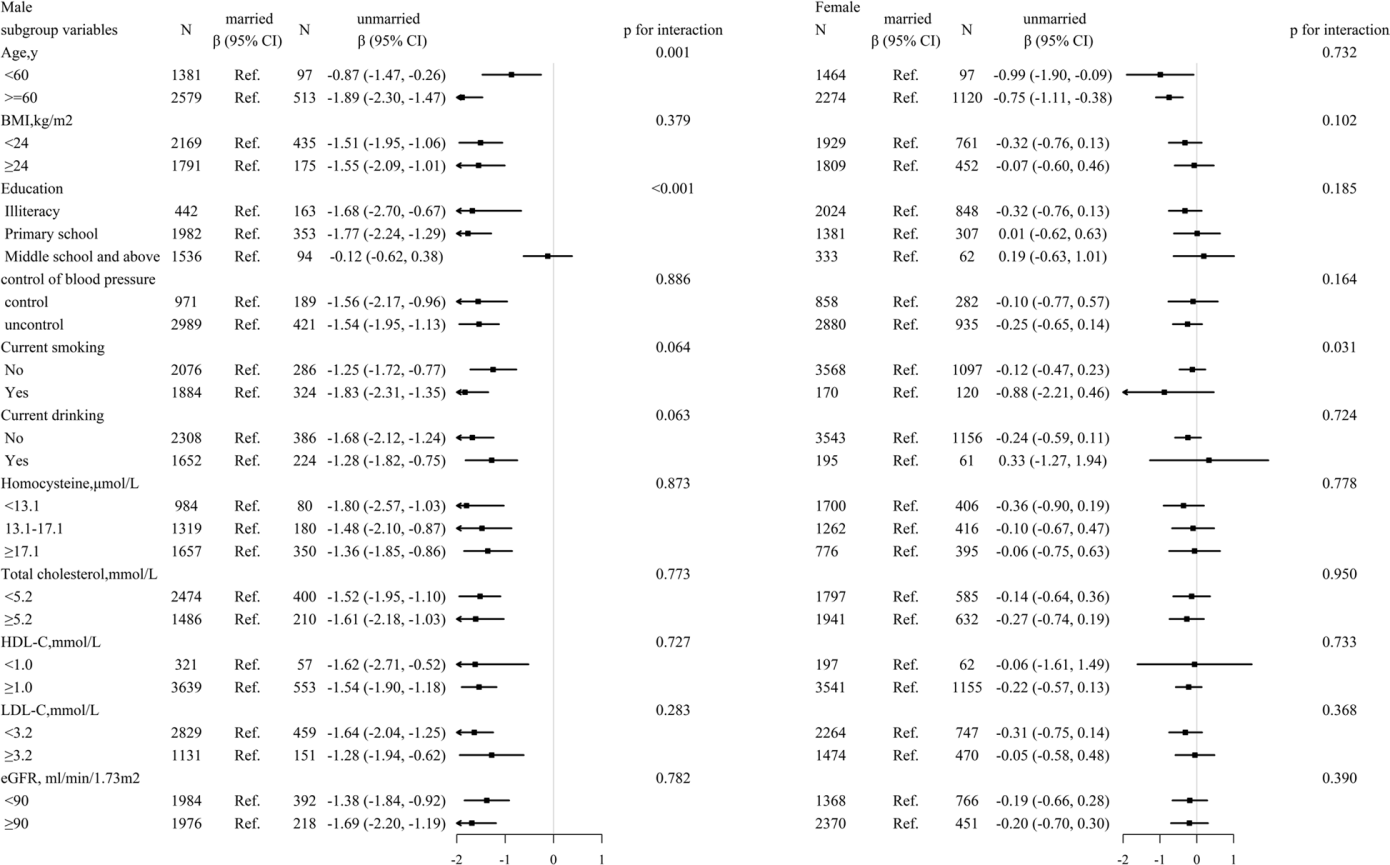
The association between different marital status and cognitive function score in various subgroups. Adjusted, if not stratified, for age, education, SBP, DBP, BMI, homocysteine, total cholesterol, triglyceride, HDL-C, LDL-C, eGFR, diabetes, coronary heart disease, standard of living, labour intensity, stress, sleep duration, antihypertensive drugs, smoking status, alcohol drinking status

Because the results showed that the link between marital status and cognitive function varies by sex (shown in Figure S1**)**, we stratified the study population into two subgroups. Further subgroup analyses were performed by several important covariables, including age, BMI, education level, control of blood pressure, current smoking, current drinking, homocysteine, total cholesterol, HDL-C, LDL-C, and eGFR. As shown in Fig. 2, except for the interaction shown in the enrolment age and education subgroup in the male multivariable-adjusted models, there were no significant interactions in any other subgroups (P for interaction > 0.05).

## Discussion

In this study of Chinese patients with hypertension, we demonstrated that an unmarried status was significantly associated with a lower MMSE score and lower MMSE subscores in five major cognitive domains. Moreover, sex was a significant effect modifier: among unmarried people, older men had lower MMSE scores, but this was not observed in women.

Previous studies have shown that married people are in better health than unmarried people, which means they also tend to live longer [8, 11]. Being single or in a bad marriage long term has many negative consequences, including leading to mental and physical harm, such as depression [23], suicide [24], high blood pressure [25], and the risk of cardiovascular disease [11] and all-cause mortality [25]. There have been some previous studies on the relationship between marital status and cognitive impairment. Similar to our research, some of these studies have shown that being widowed may be a social risk factor for cognitive impairment [9, 10, 26], but not all studies have reported this [27, 28]. Research evidence from the United States suggests that widowhood may be a risk factor for cognitive decline, and the cognitive decline of widowed elderly people will accelerate over time [9, 10]. A longitudinal study from South Korea did not come to a completely consistent conclusion. They believed that widowhood increased the risk of cognitive decline regardless of the length of widowhood [26]. The following mechanism could possibly account for the association. Having a spouse usually means that a person can obtain more care in their daily life. Spouses are often an important reason why people can obtain more understanding and support and participate in more social activities in daily life, increasing their cognitive reserve and reducing the risk of dementia, which enhances the ability to deal with neuropathic damage [29, 30]. Living alone and widowhood have been demonstrated to affect health and increase stress, both of which may increase the risk of disease and be associated with cognitive function decline [31, 32]. It has been reported that psychological stress activates hypothalamic–pituitary–adrenal axis activity and increases the levels of glucocorticoid hormones, causes damage to hippocampal structure and function [33], affects learning and memory processes [34], increases the deposition of β-amyloid peptid and τ-protein [35] in the brain and increases the incidence of cardiovascular disease [36] and hypertension [37]. All of these factors are associated with dementia [38]. In a society where marriage and childbirth are expected, people who have never married are a very vulnerable group psychologically [39], which may be one of the reasons why they have the lowest MMSE scores. Nevertheless, some longitudinal studies have come to different conclusions that widowhood does not lead to the decline of cognitive ability [27, 28]. The reasons for these different conclusions are still unclear.

Recently, a cross-sectional study involving 1,376 participants in China showed that single men had more severe cognitive impairment than married people, while similar results were not observed in single women [12]. Our results further confirm that sex may be a potential regulatory factor between marital status and cognitive function. Generally, women are more sociable and take better care of themselves when they are alone. Moreover, women tend to take more responsibility in the family for taking care of their spouses, which also means that men may receive more health benefits in marriage than women [40]. A study from the United States provided a different view. They did not find sex differences in the relationship between marital status and cognitive impairment [9]. Cultural differences may be the reason for this difference. In China, due to the influence of traditional ideas, divorce is considered disgraceful, especially for women. Therefore, women usually choose to hide their anger even in the face of marital dissatisfaction. This also means that they may benefit less from a marriage. In the West, men and women are more equal in marriage, which leads to similar benefits in marriage.

Ageing is a well-established risk factor for cognitive decline [41, 42]. However, the reason why older unmarried men had a higher risk of cognitive decline is not clear. Possible reasons include the following. With the development of the social economy and the integration of Chinese and Western cultures, young people are more accepting of divorce than elderly people [43]. Young Chinese couples are more likely to choose divorce because of dissatisfaction with their marriage, which also means that changes in marital status may have less of an impact on them. In addition, young people are more social, which is considered to have a protective effect on cognitive function [29, 30]. Finally, we know that older people tend to be in poorer health and require more care. Being in unmarried usually indicates that a person receives less care, which will accelerate cognitive decline [9, 10].

One study related to cognitive function suggests that education is an important protective factor for cognitive decline in later life [44]. Higher levels of education may increase the ability to recover from neurological effects, meaning that people with higher levels of education may need to endure greater impairments to exhibit corresponding cognitive deficits [45]. This may be one reason why male patients who were illiterate and had a primary school education had lower MMSE scores than those with a middle school education or above in our study.

Two new insights were provided in our research. First, this is the only study with hypertensive patients to investigate the correlation between different marital statuses and cognitive function. There are a large number of patients with hypertension in China. A recent study has shown that approximately 23.2% of Chinese adults suffer from hypertension [15]. Hypertension has emerged as a leading cause of cognitive impairment [13, 14], and it is necessary for us to increase our attention towards this special population. Second, our study focused on adults throughout a broad age range and demonstrated that there was a stronger link between cognitive decline and marital status in older men, rather than solely focusing on the elderly population, as previous studies have done. These findings will help health policymakers and practitioners identify subgroups that need more attention and design more effective intervention strategies to reduce the risk of dementia.

We made some efforts to make our research conclusions more convincing. In the subgroup analysis, we observed that males had worse MMSE scores than females. To better understand the differences by sex, we redivided the study population by sex and came to the following conclusion: Compared with being married, being unmarried was associated with greater cognitive impairment in Chinese hypertensive men, especially among older men, but this correlation was not observed among women. In addition, we used some blood biochemical indicators as confounding factors, which have rarely been considered in previous studies.

Several potential limitations of our study should be noted. First, with a cross-sectional design, our analysis focused on recording and identifying general associations rather than determining causality. We could not draw any causal conclusions between marital status and cognitive function from these data. Second, residual confounding factors may still have affected our results, although we adjusted for multiple potential confounding factors. For example, our study did not assess some psychosocial variables, such as depression, living alone or with a partner, the relationships between family members or the level of social support, which may affect marriage quality and cognitive function. Third, when we collected information on the marital status of the surveyed population, we only focused on their current marital status, without recording their previous marital status. That is, we do not know whether the currently married population previously experienced divorce. In addition, we do not know whether the people who had never been married had cohabiting partners. These may interfere with our research conclusions to a certain extent. Fourth, the Chinese version of the MMSE test used in our study could only provide a rough assessment of cognitive function [46]. Thus, this tool may not be able to detect subtle changes in cognitive function.

## Conclusion

In summary, this cross-sectional study showed that being unmarried was an extremely important but neglected social risk factor for cognitive function. Sex was a strong effect modifier: being unmarried was correlated with a higher risk of cognitive decline than being married in Chinese hypertensive men, especially among older men, but this correlation was not observed among women. Moreover, never married men showed poorer cognitive function than those who were divorced or widowed. However, because the sample size of the never-married population in this study was relatively small, this conclusion still needs to be confirmed by some larger sample size studies with other populations in the future.

## Supplementary Information

**Additional file 1.**

## Data Availability

The datasets generated and analysed during the current study are not publicly available because this study is still on-going and the follow-up is not finished, but they are available from the corresponding author on reasonable request.

## Abbreviations

MMSE: Chines adapted MMSE (CAMSE)
SD: Standard deviation
SBP: Systolic blood pressure
DBP: Diastolic blood pressure
eGFR: Estimated glomerular filtration rate
CKD-EPI: Chronic Kidney Disease Epidemiology Collaboration
BMI: Body mass index
HDL-C: High-density lipoprotein cholesterol
LDL-C: Low-density lipoprotein cholesterol
CI: Confidence interval
CHD: Coronary heart disease
