# Supplementary material for: Association of marital status with cognitive function in Chinese hypertensive patients: a cross-sectional study
Source: BMC Psychiatry. 2022 Jul 27;22:504. doi: 10.1186/s12888-022-04159-9 (PMC9327272; doi:10.1186/s12888-022-04159-9)
Supplement: Supplementary file 1 — Additional file 1. [file 12888_2022_4159_MOESM1_ESM.docx]

**Table S1** Baseline characteristics of study participants.

|  |  | Marital status | | | |  | |
| --- | --- | --- | --- | --- | --- | --- | --- |
| Characteristics | Total | married | never married | divorced | widowed | | P-value |
| N | 9525 | 7698 | 96 | 52 | 1679 | |  |
| Male,N(%) | 4570 (48.0) | 3960 (51.4) | 92 (95.8) | 37 (71.2) | 481 (28.6) | | <0.001 |
| Age,years | 63.7 ± 9.8 | 62.1 ± 9.3 | 59.4 ± 11.0 | 58.6 ± 10.1 | 71.4 ± 8.2 | | <0.001 |
| BMI,kg/m² | 23.6 ± 3.5 | 23.8 ± 3.5 | 23.1 ± 4.0 | 23.7 ± 3.8 | 22.7 ± 3.4 | | <0.001 |
| SBP,mmHg | 147.2 ± 17.5 | 146.8 ± 17.2 | 144.3 ± 18.9 | 145.5 ± 19.4 | 149.4 ± 18.3 | | <0.001 |
| DBP,mmHg | 89.0 ± 10.8 | 89.8 ± 10.6 | 90.4 ± 12.4 | 93.2 ± 11.9 | 85.0 ± 10.6 | | <0.001 |
| Current smoking, N(%) | 2498 (26.2) | 2054 (26.7) | 51 (53.1) | 20 (38.5) | 373 (22.2) | | <0.001 |
| Current drinking, N(%) | 2132 (22.4) | 1847 (24.0) | 30 (31.2) | 21 (40.4) | 234 (13.9) | | <0.001 |
| MMSE | 22.1 ± 6.4 | 22.8 ± 6.1 | 21.8 ± 6.3 | 24.2 ± 5.1 | 18.4 ± 6.6 | | <0.001 |
| Laboratory results |  |  |  |  |  | |  |
| Homocysteine,umol/L | 17.9 ± 11.1 | 17.6 ± 11.0 | 21.6 ± 13.0 | 20.1 ± 13.8 | 18.7 ± 11.1 | | <0.001 |
| Total cholesterol,mmol/L | 5.1 ± 1.1 | 5.1 ± 1.1 | 5.1 ± 1.1 | 5.0 ± 0.8 | 5.2 ± 1.1 | | 0.231 |
| Triglyceride,mmol/L | 1.8 ± 1.3 | 1.9 ± 1.4 | 1.7 ± 1.0 | 1.8 ± 1.2 | 1.6 ± 1.0 | | <0.001 |
| LDL-C,mmol/L | 2.9 ± 0.8 | 2.9 ± 0.8 | 3.0 ± 0.9 | 2.9 ± 0.6 | 2.9 ± 0.8 | | 0.92 |
| HDL-C,mmol/L | 1.5 ± 0.4 | 1.5 ± 0.4 | 1.5 ± 0.4 | 1.4 ± 0.4 | 1.6 ± 0.4 | | <0.001 |
| eGFR,ml/min/1.73m² | 86.3 ± 19.6 | 87.9 ± 19.1 | 88.4 ± 22.8 | 89.1 ± 19.9 | 79.0 ± 19.8 | | <0.001 |
| Education,N(%) | | | | | | | <0.001 |
| Illiteracy | 3477 (36.5) | 2466 (32.0) | 22 (22.9) | 13 (25.0) | 976 (58.1) | |  |
| Primary school | 4023 (42.2) | 3363 (43.7) | 61 (63.5) | 25 (48.1) | 574 (34.2) | |  |
| Middle school and above | 2025 (21.3) | 1869 (24.3) | 13 (13.5) | 14 (26.9) | 129 (7.7) | |  |
| Standard of living, N(%) |  |  |  |  |  | | <0.001 |
| Better | 1246 (13.1) | 1003 (13.0) | 6 (6.2) | 2 (3.8) | 235 (14.0) | |  |
| General | 6491 (68.1) | 5369 (69.7) | 41 (42.7) | 33 (63.5) | 1048 (62.4) | |  |
| Poorer | 1788 (18.8) | 1326 (17.2) | 49 (51.0) | 17 (32.7) | 396 (23.6) | |  |
| Labour intensity, N(%) |  |  |  |  |  | | <0.001 |
| Light | 5310 (55.7) | 4129 (53.6) | 58 (60.4) | 30 (57.7) | 1093 (65.1) | |  |
| Medium | 2219 (23.3) | 1895 (24.6) | 21 (21.9) | 13 (25.0) | 290 (17.3) | |  |
| Heavy | 1996 (21.0) | 1674 (21.7) | 17 (17.7) | 9 (17.3) | 296 (17.6) | |  |
| Stress, N(%) |  |  |  |  |  | | 0.371 |
| Rarely | 6408 (67.3) | 5182 (67.3) | 55 (57.3) | 33 (63.5) | 1138 (67.8) | |  |
| Sometimes | 2294 (24.1) | 1854 (24.1) | 31 (32.3) | 16 (30.8) | 393 (23.4) | |  |
| Always | 823 (8.6) | 662 (8.6) | 10 (10.4) | 3 (5.8) | 148 (8.8) | |  |
| Sleep duration, h, N(%) |  |  |  |  |  | | <0.001 |
| <5 | 395 (4.1) | 290 (3.8) | 2 (2.1) | 4 (7.7) | 99 (5.9) | |  |
| ≥5 to <8 | 4958 (52.1) | 4096 (53.2) | 42 (43.8) | 29 (55.8) | 791 (47.1) | |  |
| ≥8 | 4172 (43.8) | 3312 (43.0) | 52 (54.2) | 19 (36.5) | 789 (47.0) | |  |
| History of disease |  |  |  |  |  | |  |
| Diabetes, N(%) | 1726 (18.1) | 1403 (18.2) | 21 (21.9) | 9 (17.3) | 293 (17.5) | | 0.682 |
| CHD, N(%) | 533 (5.6) | 407 (5.3) | 7 (7.3) | 3 (5.8) | 116 (6.9) | | 0.06 |
| Medication use, N(%) |  |  |  |  |  | |  |
| Antihypertensive drugs | 5798 (60.9) | 4617 (60.0) | 49 (51.0) | 29 (55.8) | 1103 (65.7) | | <0.001 |
| Glucose-lowering drugs | 447 (4.7) | 368 (4.8) | 5 (5.2) | 1 (1.9) | 73 (4.3) | | 0.676 |
| Lipoprotein-lowering drugs | 258 (2.7) | 219 (2.8) | 2 (2.1) | 0 (0.0) | 37 (2.2) | | 0.289 |

Note: Data are expressed as mean ± SD and numbers (percentage) as appropriate. Abbreviations: BMI, body mass index; SBP, systolic blood pressure; DBP, diastolic blood pressure; MMSE, Mini‐Mental State Examination; LDL-C, low-density lipoprotein cholesterol; HDL-C, high-density lipoprotein cholesterol; eGFR, estimated glomerular filtration rate; CHD, coronary heart disease.

**Table S2** Regression coefficients (95% CIs) of orientation total score according to marital status.

|  |  |  | Adjusted model |  |
| --- | --- | --- | --- | --- |
| Marital status | N | Mean+SD | orientation β (95% CI) | p value |
| Total |  |  |  |  |
| married | 7698 | 8.3 ± 2.1 | Ref. |  |
| never married | 96 | 8.4 ± 2.2 | -0.50 (-0.85, -0.15) | 0.005 |
| divorced | 52 | 8.9 ± 1.6 | 0.15 (-0.32, 0.62) | 0.521 |
| widowed | 1679 | 7.0 ± 2.6 | -0.26 (-0.35, -0.16) | <0.001 |
| Marital status |  |  |  |  |
| married | 7698 | 8.3 ± 2.1 | Ref. |  |
| unmarried | 1827 | 7.1 ± 2.6 | -0.26 (-0.35, -0.16) | <0.001 |
| Male |  |  |  |  |
| married | 3960 | 9.2 ± 1.3 | Ref. |  |
| never married | 92 | 8.4 ± 2.2 | -0.53 (-0.79, -0.27) | <0.001 |
| divorced | 37 | 9.2 ± 1.1 | 0.23 (-0.18, 0.64) | 0.269 |
| widowed | 481 | 8.4 ± 2.0 | -0.31 (-0.44, -0.19) | <0.001 |
| Marital status |  |  |  |  |
| married | 3960 | 9.2 ± 1.3 | Ref. |  |
| unmarried | 610 | 8.4 ± 2.0 | -0.31 (-0.42, -0.20) | <0.001 |
| Female |  |  |  |  |
| married | 3738 | 7.4 ± 2.4 | Ref. |  |
| never married | 4 | 7.5 ± 2.9 | 0.72 (-1.56, 3.01) | 0.535 |
| divorced | 15 | 8.1 ± 2.2 | -0.36 (-1.39, 0.66) | 0.491 |
| widowed | 1198 | 6.4 ± 2.6 | -0.11 (-0.26, 0.04) | 0.153 |
| Marital status |  |  |  |  |
| married | 3738 | 7.4 ± 2.4 | Ref. |  |
| unmarried | 1217 | 6.5 ± 2.6 | -0.11 (-0.26, 0.04) | 0.142 |

Adjusted model was adjusted for sex (sex was adjusted only in the total sample), age, education, SBP, DBP, BMI, homocysteine, total cholesterol, triglyceride, HDL-C, LDL-C, eGFR, diabetes, coronary heart disease, standard of living, labour intensity, stress, sleep duration, antihypertensive drugs, smoking status, alcohol drinking status.

**Table S3** Regression coefficients (95% CIs) of immediate memory total score according to marital status

|  |  |  | Adjusted model |  |
| --- | --- | --- | --- | --- |
| Marital status | N | Mean+SD | immediate memory β (95% CI) | p value |
| Total |  |  |  |  |
| married | 7698 | 2.4 ± 1.0 | Ref. |  |
| never married | 96 | 2.3 ± 1.1 | -0.21 (-0.41, -0.01) | 0.037 |
| divorced | 52 | 2.5 ± 0.9 | 0.00 (-0.26, 0.26) | 0.992 |
| widowed | 1679 | 1.9 ± 1.2 | -0.12 (-0.18, -0.07) | <0.001 |
| Marital status |  |  |  |  |
| married | 7698 | 2.4 ± 1.0 | Ref. |  |
| unmarried | 1827 | 2.0 ± 1.2 | -0.13 (-0.18, -0.07) | <0.001 |
| Male |  |  |  |  |
| married | 3960 | 2.6 ± 0.8 | Ref. |  |
| never married | 92 | 2.2 ± 1.2 | -0.21 (-0.38, -0.03) | 0.021 |
| divorced | 37 | 2.6 ± 0.9 | 0.06 (-0.21, 0.33) | 0.668 |
| widowed | 481 | 2.2 ± 1.1 | -0.12 (-0.21, -0.04) | 0.004 |
| Marital status |  |  |  |  |
| married | 3960 | 2.6 ± 0.8 | Ref. |  |
| unmarried | 610 | 2.2 ± 1.1 | -0.12 (-0.20, -0.05) | 0.001 |
| Female |  |  |  |  |
| married | 3738 | 2.3 ± 1.1 | Ref. |  |
| never married | 4 | 2.5 ± 1.0 | 0.07 (-1.13, 1.28) | 0.905 |
| divorced | 15 | 2.4 ± 1.0 | -0.13 (-0.67, 0.41) | 0.629 |
| widowed | 1198 | 1.9 ± 1.2 | -0.08 (-0.16, -0.00) | 0.038 |
| Marital status |  |  |  |  |
| married | 3738 | 2.3 ± 1.1 | Ref. |  |
| unmarried | 1217 | 1.9 ± 1.2 | -0.08 (-0.16, -0.01) | 0.035 |

Adjusted model was adjusted for sex (sex was adjusted only in the total sample), age, education, SBP, DBP, BMI, homocysteine, total cholesterol, triglyceride, HDL-C, LDL-C, eGFR, diabetes, coronary heart disease, standard of living, labour intensity, stress, sleep duration, antihypertensive drugs, smoking status, alcohol drinking status.

**Table S4** Regression coefficients (95% CIs) of attention and computation total score according to marital status.

|  |  |  | Adjusted model |  |
| --- | --- | --- | --- | --- |
| Marital status | N | Mean+SD | attention and computation β (95% CI) | p value |
| Total |  |  |  |  |
| married | 7698 | 2.9 ± 1.9 | Ref. |  |
| never married | 96 | 2.4 ± 1.9 | -0.85 (-1.16, -0.54) | <0.001 |
| divorced | 52 | 3.3 ± 1.9 | 0.03 (-0.38, 0.45) | 0.872 |
| widowed | 1679 | 1.8 ± 1.9 | -0.17 (-0.26, -0.09) | 0.001 |
| Marital status |  |  |  |  |
| married | 7698 | 2.9 ± 1.9 | Ref. |  |
| unmarried | 1827 | 1.9 ± 1.9 | -0.21 (-0.29, -0.13) | <0.001 |
| Male |  |  |  |  |
| married | 3960 | 3.6 ± 1.6 | Ref. |  |
| never married | 92 | 2.4 ± 2.0 | -0.90 (-1.21, -0.58) | <0.001 |
| divorced | 37 | 3.3 ± 1.9 | -0.14 (-0.62, 0.35) | 0.588 |
| widowed | 481 | 2.5 ± 1.9 | -0.51 (-0.66, -0.37) | <0.001 |
| Marital status |  |  |  |  |
| married | 3960 | 3.6 ± 1.6 | Ref. |  |
| unmarried | 610 | 2.6 ± 1.9 | -0.55 (-0.68, -0.42) | <0.001 |
| Female |  |  |  |  |
| married | 3738 | 2.1 ± 1.9 | Ref. |  |
| never married | 4 | 1.2 ± 1.3 | -0.95 (-2.67, 0.76) | 0.277 |
| divorced | 15 | 3.2 ± 2.1 | 0.20 (-0.57, 0.98) | 0.603 |
| widowed | 1198 | 1.5 ± 1.8 | 0.04 (-0.08, 0.15) | 0.536 |
| Marital status |  |  |  |  |
| married | 3738 | 2.1 ± 1.9 | Ref. |  |
| unmarried | 1217 | 1.6 ± 1.8 | 0.03 (-0.08, 0.15) | 0.534 |

Adjusted model was adjusted for sex (sex was adjusted only in the total sample), age, education, SBP, DBP, BMI, homocysteine, total cholesterol, triglyceride, HDL-C, LDL-C, eGFR, diabetes, coronary heart disease, standard of living, labour intensity, stress, sleep duration, antihypertensive drugs, smoking status, alcohol drinking status.

**Table S5** Regression coefficients (95% CIs) of recall total score according to marital status.

|  |  |  | Adjusted model |  |
| --- | --- | --- | --- | --- |
| Marital status | N | Mean+SD | recall β (95% CI) | p value |
| Total |  |  |  |  |
| married | 7698 | 2.0 ± 1.2 | Ref. |  |
| never married | 96 | 1.8 ± 1.3 | -0.16 (-0.40, 0.07) | 0.173 |
| divorced | 52 | 2.1 ± 1.2 | -0.02 (-0.34, 0.29) | 0.893 |
| widowed | 1679 | 1.5 ± 1.3 | -0.15 (-0.22, -0.09) | <0.001 |
| Marital status |  |  |  |  |
| married | 7698 | 2.0 ± 1.2 | Ref. |  |
| unmarried | 1827 | 1.5 ± 1.3 | -0.15 (-0.21, -0.08) | <0.001 |
| Male |  |  |  |  |
| married | 3960 | 2.1 ± 1.1 | Ref. |  |
| never married | 92 | 1.8 ± 1.3 | -0.19 (-0.42, 0.04) | 0.113 |
| divorced | 37 | 2.0 ± 1.2 | -0.05 (-0.40, 0.31) | 0.797 |
| widowed | 481 | 1.5 ± 1.3 | -0.23 (-0.34, -0.12) | <0.001 |
| Marital status |  |  |  |  |
| married | 3960 | 2.1 ± 1.1 | Ref. |  |
| unmarried | 610 | 1.6 ± 1.3 | -0.21 (-0.31, -0.11) | <0.001 |
| Female |  |  |  |  |
| married | 3738 | 1.9 ± 1.2 | Ref. |  |
| never married | 4 | 3.0 ± 0.0 | 1.11 (-0.24, 2.46) | 0.107 |
| divorced | 15 | 2.2 ± 1.3 | 0.01 (-0.60, 0.61) | 0.976 |
| widowed | 1198 | 1.4 ± 1.3 | -0.09 (-0.18, -0.00) | 0.043 |
| Marital status |  |  |  |  |
| married | 3738 | 1.9 ± 1.2 | Ref. |  |
| unmarried | 1217 | 1.5 ± 1.3 | -0.08 (-0.17, 0.00) | 0.057 |

Adjusted model was adjusted for sex (sex was adjusted only in the total sample), age, education, SBP, DBP, BMI, homocysteine, total cholesterol, triglyceride, HDL-C, LDL-C, eGFR, diabetes, coronary heart disease, standard of living, labour intensity, stress, sleep duration, antihypertensive drugs, smoking status, alcohol drinking status.

**Table S6** Regression coefficients (95% CIs) of language total score according to marital status.

|  |  |  | Adjusted model |  |
| --- | --- | --- | --- | --- |
| Marital status | N | Mean+SD | language β (95% CI) | p value |
| Total |  |  |  |  |
| married | 7698 | 7.2 ± 1.7 | Ref. |  |
| never married | 96 | 7.0 ± 1.8 | -0.46 (-0.71, -0.21) | 0.003 |
| divorced | 52 | 7.5 ± 1.4 | 0.00 (-0.33, 0.33) | 0.990 |
| widowed | 1679 | 6.1 ± 1.7 | -0.11 (-0.18, -0.04) | 0.003 |
| Marital status |  |  |  |  |
| married | 7698 | 7.2 ± 1.7 | Ref. |  |
| unmarried | 1827 | 6.2 ± 1.7 | -0.13 (-0.19, -0.06) | <0.001 |
| Male |  |  |  |  |
| married | 3960 | 7.9 ± 1.4 | Ref. |  |
| never married | 92 | 7.0 ± 1.8 | -0.48 (-0.73, -0.23) | <0.001 |
| divorced | 37 | 7.5 ± 1.3 | -0.09 (-0.48, 0.29) | 0.640 |
| widowed | 481 | 6.8 ± 1.9 | -0.35 (-0.47, -0.23) | <0.001 |
| Marital status |  |  |  |  |
| married | 3960 | 7.9 ± 1.4 | Ref. |  |
| unmarried | 610 | 6.9 ± 1.9 | -0.36 (-0.46, -0.25) | <0.001 |
| Female |  |  |  |  |
| married | 3738 | 6.4 ± 1.6 | Ref. |  |
| never married | 4 | 6.0 ± 0.8 | -0.41 (-1.80, 0.98) | 0.562 |
| divorced | 15 | 7.4 ± 1.7 | 0.16 (-0.46, 0.79) | 0.607 |
| widowed | 1198 | 5.9 ± 1.5 | 0.02 (-0.07, 0.12) | 0.590 |
| Marital status |  |  |  |  |
| married | 3738 | 6.4 ± 1.6 | Ref. |  |
| unmarried | 1217 | 5.9 ± 1.5 | 0.03 (-0.06, 0.12) | 0.569 |

Adjusted model was adjusted for sex (sex was adjusted only in the total sample), age, education, SBP, DBP, BMI, homocysteine, total cholesterol, triglyceride, HDL-C, LDL-C, eGFR, diabetes, coronary heart disease, standard of living, labour intensity, stress, sleep duration, antihypertensive drugs, smoking status, alcohol drinking status.


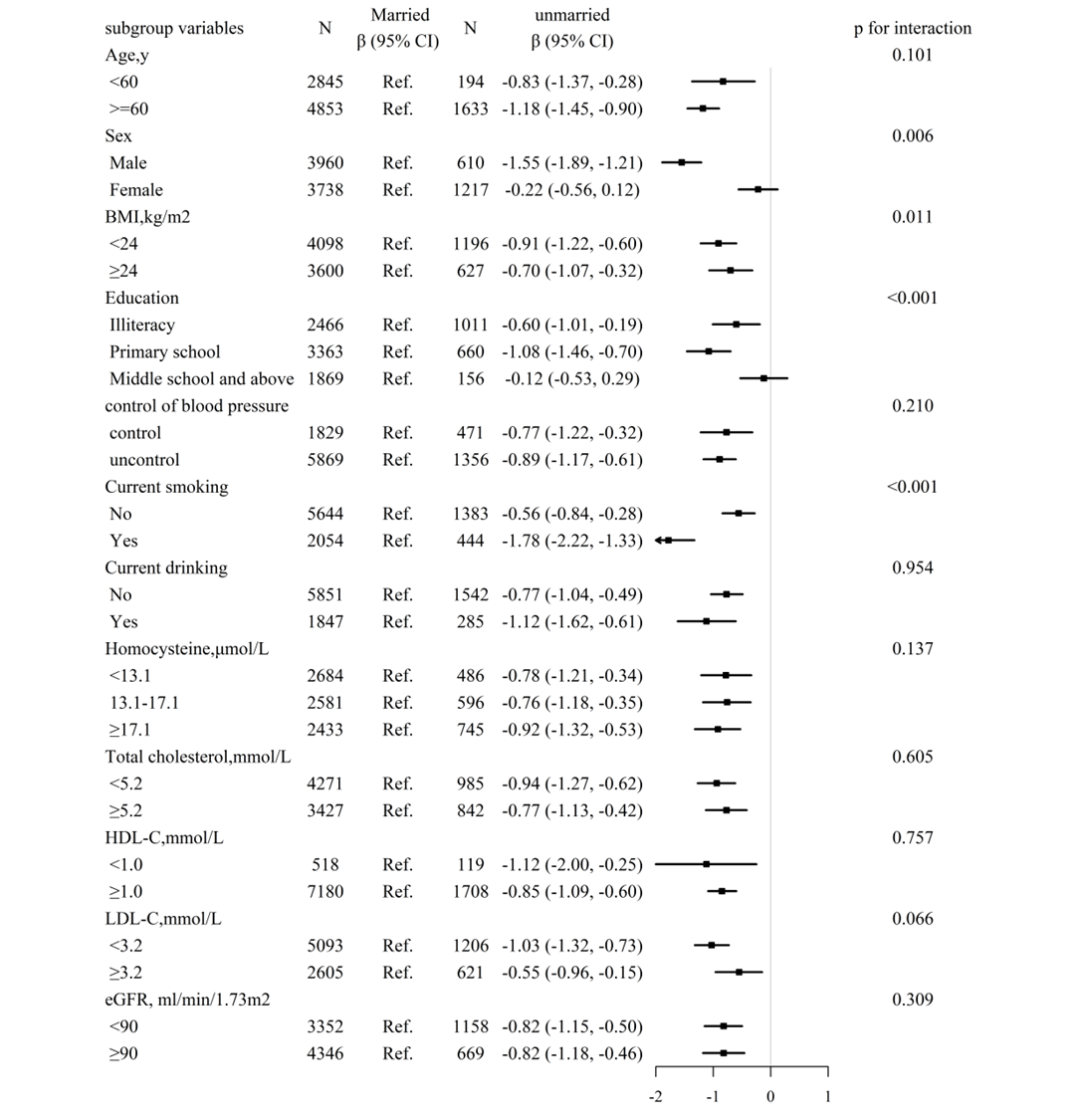


**Figure S1** The association between different marital status and cognitive function score in various subgroups *. *Adjusted, if not stratified, for sex, age, education, SBP, DBP, BMI, homocysteine, total cholesterol, triglyceride, HDL-C, LDL-C, eGFR, diabetes, coronary heart disease, standard of living, labour intensity, stress, sleep duration, antihypertensive drugs, smoking status, alcohol drinking status.
